# Natural Formulations Based on *Olea europaea* L. Fruit Extract for the Topical Treatment of HSV-1 Infections

**DOI:** 10.3390/molecules27134273

**Published:** 2022-07-02

**Authors:** Stefania Cometa, Carla Zannella, Francesco Busto, Anna De Filippis, Gianluigi Franci, Massimiliano Galdiero, Elvira De Giglio

**Affiliations:** 1 Jaber Innovation s.r.l., Via Calcutta 8, 00144 Rome, Italy; stefania.cometa@jaber.it; 2 Department of Experimental Medicine, Università degli Studi della Campania Luigi Vanvitelli, 80138 Naples, Italy; carla.zannella@unicampania.it (C.Z.); anna.defilippis@unicampania.it (A.D.F.); 3 Department of Chemistry, University of Bari, Via Orabona 4, 70126 Bari, Italy; f.busto3@studenti.uniba.it; 4 Department of Medicine, Surgery and Dentistry “Scuola Medica Salernitana”, University of Salerno, 84081 Baronissi, Italy; gfranci@unisa.it; 5 INSTM, National Consortium of Materials Science and Technology, Via G. Giusti 9, 50121 Florence, Italy

**Keywords:** olive fruit extract, polyphenol, antiviral, HSV-1, topical formulations, Franz cell, acyclovir-resistant strain

## Abstract

In the present study, a hydroxytyrosol-rich *Olea europaea* L. fruit extract (OFE) was added to three thoroughly green formulations—hydrogel, oleogel, and cream—in order to evaluate their antiviral activity against HSV-1. The extract was characterized by different analytical techniques, i.e., FT-IR, XPS, and TGA. HPLC analyses were carried out to monitor the content and release of hydroxytyrosol in the prepared formulations. The total polyphenol content and antioxidant activity were investigated through Folin–Ciocâlteu’s reagent, DPPH, and ABTS assays. The ability of the three formulations to convey active principles to the skin was evaluated using a Franz cell, showing that the number of permeated polyphenols in the hydrogel (272.1 ± 1.8 GAE/g) was significantly higher than those in the oleogel and cream (174 ± 10 and 179.6 ± 2 GAE/g, respectively), even if a negligible amount of hydroxytyrosol crossed the membrane for all the formulations. The cell viability assay indicated that the OFE and the three formulations were not toxic to cultured Vero cells. The antiviral activity tests highlighted that the OFE had a strong inhibitory effect against HSV-1 with a 50% inhibitory concentration (IC50) at 25 µg/mL, interfering directly with the viral particles. Among the three formulations, the hydrogel exhibited the highest antiviral activity also against the acyclovir-resistant strain.

## 1. Introduction

Nowadays, due to the current pandemic, viral infections are one of the most dangerous problems affecting the world. The main causes of growing viral infections arise from the consequences of globalization such as demographic increases, migratory flows, low hygiene standards in developing countries, the high international circulation of people and goods, densely populated urban agglomerations, the destruction of ecosystems, and pollution. Herpes simplex virus type 1 (HSV-1) is a common pathogen whose infection is characterized by the formation of epidermal lesions in and around the mouth and/or nose, as well as in other areas of the body, with frequent relapses [1]. In some cases, i.e., in immunocompromised patients, HSV-1 can cause severe complications such as encephalitis, keratitis [2,3], and severe and chronic neurological diseases of the nervous system [4]. Current antiviral drugs used to treat HSV-1 include acyclovir, ganciclovir, valaciclovir, and penciclovir. These drugs prevent viruses from multiplying and, as a result, hinder the emergence of severe symptoms of herpes and reduce the chances of transmission. On the other hand, up to 10% of immunosuppressed individuals may develop HSV-1 strains that are resistant to the most commonly used antivirals. Moreover, the proliferation of infections causes the onset of drug-resistant phenomena [5].

In this scenario, the research of drugs of a botanical origin might represent valid future treatments for herpetic diseases, based on their ability to conjugate high safety and efficacy [6,7]. In this respect, polyphenol-rich vegetal extracts can be considered promising alternatives to synthetic drugs. Indeed, different studies have demonstrated that polyphenols display anti-infective properties, avoiding serious toxicity effects, accompanied by remarkable antioxidant properties, which are useful for the prevention or treatment of oxidative stress-correlated diseases [8].

The olive tree (*Olea europaea* L.) has been one of the most important fruit trees in Mediterranean countries for over 7000 years. The disease-preventing properties of olives and the oil obtained through olive pressing are due to the presence of several bioactive compounds, such as unsaturated fatty acids (i.e., oleic and linoleic acids) and other minor but fundamental compounds, such as polyphenols. In particular, olive fruit extract (OFE) is a rich source of phenolic molecules, such as hydroxytyrosol, tyrosol, and their secoiridoid derivatives (oleuropein, oleuropein aglycone, and oleanolic acid dialdehydes); verbascoside; lignans; and flavonoids [9], which could act in synergy. Different studies have reported the employment of olive leaf extract (OLE) for the treatment of HSV-1 infections [10,11,12]. Ben Amor I. et al. demonstrated the strong anti-HSV-1 effect of OLE by direct virucidal action at a 50% inhibitory concentration (IC50) of 100 µg/mL. Moreover, the extract was able to prevent the replicative mechanisms by acting inside the infected cell at an IC50 of 200 µg/mL [7]. For all these positive in vitro effects, the impact of a 2% OLE cream was recently analyzed on the healing of HSV-1 infections and compared with a 5% acyclovir cream in 62 patients enrolled in two groups [10]. The results demonstrated that the healing time of the OLE group was significantly shorter than that of the acyclovir group and that the patients belonging to the first group developed less severe and lasting clinical symptoms. On the other hand, based on our knowledge, the employment of OFE for herpetic diseases is not well-documented, probably due to its minor water solubility. First, we analyzed the antiherpetic effect of a panel of olive extracts, and OFE demonstrated superior antiviral properties against HSV-1 than different OLEs, particularly in its oleuropein component.

For this reason, in the present study, we used OFE as an initial source to obtain three different formulations for topical application on mucosae and skin for the treatment of HSV-1 infection through thoroughly green strategies. A detailed analytical characterization of OFE has been carried out using Fourier Transform Infrared Spectroscopy (FT-IR) in Attenuated Total Reflectance (ATR), X-ray Photoelectron Spectroscopy (XPS), and Thermo-Gravimetric Analysis (TGA). The hydroxytyrosol content has been monitored by HPLC analyses.

The basic idea of this study relies on designing totally bio-based formulations, with eco-certified, vegetable, or naturally derived raw materials. The OFE-loaded formulations consisted of an aqueous gel (called hydrogel), an oily gel (called oleogel), and a cream. Release studies have been performed using HPLC for monitoring the hydroxytyrosol [13] after a preliminary extraction of the molecule in ethyl acetate. The total polyphenol content (TPC) and antioxidant activity have been investigated in vitro through Folin–Ciocâlteu’s phenol reagent, DPPH, and ABTS assays.

An antiviral topical formulation, in order to exert the greatest effect, should release the active ingredients at therapeutic concentrations in the basal epidermal cells, which are the initial port of entry for the virus [14]. Consequently, skin permeation is fundamental for the success of the therapy against HSV-1 using a topical formulation. In this respect, permeation studies using a Franz cell, have been carried out for all the proposed OFE-loaded formulations. Another important parameter to consider for the production of antiviral topical formulations is that they should be non-toxic for cells and should not cause any side effects. All the formulations in this study did not exhibit any toxicity on the cell system used in the antiviral experiments. We investigated the activity of the OFE-containing formulations through plaque assays against HSV-1 and its acyclovir-resistant strain by showing a strong antiherpetic activity at very low concentrations for two formulations (i.e., hydrogel and cream). The effects could be ascribed to the inhibition of the early phases of infection by interfering directly with the viral particles. No effect has been observed on the cell bilayer, indicating that the tested formulations could have a specific action only on the viral membrane. Moreover, the Franz cell tests evidenced a hydroxytyrosol and total polyphenol permeation from the hydrogel, which was significantly higher than that of the other two formulations. Altogether, these results indicated that the hydrogel-based topical formulation had the most suitable characteristics to treat HSV-1 infections.

## 2. Results and Discussion

### 2.1. Initial Antiviral Screening of Olea Europaea Extracts

**Figure 1:**
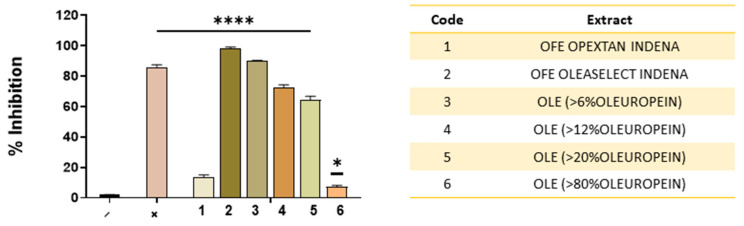
Screening of olive extracts. Co-treatment assay: cells were treated with each extract (200 μg/mL) and infected with HSV-1 at the same time. Negative control (−) indicates infected but non-treated cells, whereas positive control (+) refers to Greco grape cane extract at 10 μg/mL [7]. Statistical analyses were determined by ANOVA with Dunnett’s test for multiple comparisons. Significances are referred to as the negative control (−). **** *p* < 0.0001; * *p* < 0.021.

Before starting the workflow of the present study, we chose to first investigate which type of olive extract had the greatest antiherpetic activity. We screened six different olive extracts (Figure 1): the first two (indicated as 1 and 2) belong to OFE and they are Opextan^®^ (Indena, S.p.A., Milan, Italy) and Oleaselect (Indena). The other four are OLE (indicated as 3, 4, 5, and 6) and they exhibit different amounts of oleuropein ranging from 6% to 80%. A preliminary screening was very useful for establishing which extract could be the best candidate for the present study. Vero cells were simultaneously treated with each extract at 200 μg/mL and infected with the virus. After 1 h incubation, required for the viral adsorption on the host cell, non-attached viruses were removed by a gentle wash with a saline buffer (PBS) and the cell monolayer was overlaid with CMC supplemented in the culture medium (DMEM). After 48 h post-infection (hpi), the viral plaques were stained and used to determine the inhibition percentage of infectivity compared to non-treated cells (CTRL −). The results reported in Figure 1 indicated that the OFE Oleaselect (Indena) had a higher antiviral activity compared to both OFE Opextan^®^ (Indena) and the OLE extracts.

It is worth noting that as the quantity of oleuropein increased, reduced antiherpetic activity was observed. Altogether, these data suggested using the OFE Oleaselect (Indena) extract for further analyses. For simplicity, the OFE Oleaselect (Indena) extract is indicated as OFE throughout the text.

### 2.2. Chemical–Physical Characterization of the OFE

**Figure 2:**
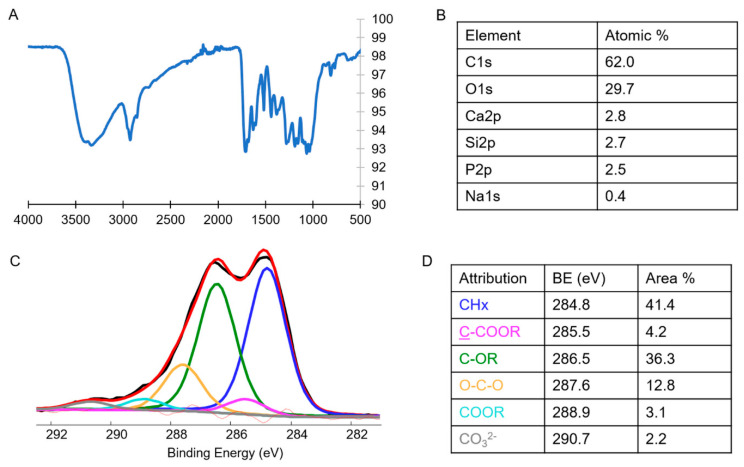
FT-IR/ATR spectrum (**A**), XPS surface atomic composition (**B**), XPS C1s signal curve fitting (**C**), and curve-fitting data (**D**) of OFE. The maximum error on the BE values was equal to ± 0.2 eV.

In Figure 2A, the FT-IR/ATR spectrum of the OFE is reported. The main peaks appeared at 3329 cm^−1^ (OH stretching, typical of polyphenol-rich extracts, as well as in mono- or polysaccharides), 2926 and 2855 cm^−1^ (C H stretching), 1707 and 1683 cm^−1^ (C=O stretching of ester or carboxylic groups, due to triglycerides and/or fatty acids), 1629 cm^−1^ (conjugated carbonyl), 1517 and 1440 cm^−1^ (bending vibrations of aliphatic groups), and the region in the range 1200–850 cm^−1^ linked to the carbohydrate fractions, as reported in the literature [15]. In Figure 2B, the XPS atomic surface composition relevant to the OFE is reported. In Figure 2C, the OFE C1s signal and the relevant curve fitting are shown. The contributions and their binding energy (BE) values together with the corrected area percentages are reported in Figure 2D.

From the C1s curve fitting, the presence of carboxylic acid or ester moieties was evident as well as alcoholic and/or ether functionalities. The presence of polysaccharide fractions, observed also in the FT-IR analysis, was confirmed by the occurrence of hemiacetal groups, falling at 287.8 eV. Overall, the FT-IR and XPS analyses revealed that the OFE was a mixture of a variety of different chemical species, as expected.

**Figure 3:**
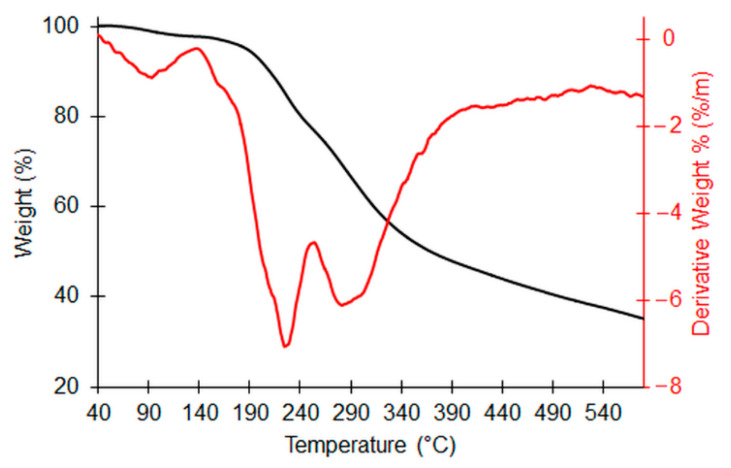
TGA (black line) and DTGA (red line) of OFE.

This finding was also confirmed by TGA analysis, reported in Figure 3. Indeed, the thermal events were manifold, as can be seen from the derivative thermogram (DGTA), indicating that this dry extract is certainly a mixture of several organic compounds. However, two main peaks can be detected at 225.8 and 304.6 °C corresponding to pyrolytic events accounting for 13.8 and 24.7% of the weight-loss percentage, respectively. We hypothesized that hydroxytyrosol thermal decomposition fell in this second stage (even if it could not be excluded in the co-presence of other species decomposing in the same temperature range) since, in the literature, its decomposition was found to occur between 262.8 and 409.7 °C, with a maximum at 305.2 °C [16]. The residue at 800 °C is equal to 15.4%, which is indicative of the presence of inorganic salts or species (as also confirmed by the surface chemical composition reported in Figure 2B). The T_onset_ (i.e., the temperature corresponding to the 1% weight loss) was found to be 176.3 °C, thus indicating the high thermostability of the natural extract. The water and/or volatile content was equal to 0.1%.

As far as the TPC evaluation is concerned, the value expressed as mg of gallic acid equivalent to GAE/g of dry extract was 430 GAE/g. This value was significantly higher than that obtained for the OLE reported by Fabiano et al. [17], for other olive leaf extracts obtained from different cultivars screened by Ozcan et al. [18], or for olive byproduct extracts studied by Cádiz-Gurrea et al. [19].

The investigated olive fruit extract evidenced a high polyphenol content as also evidenced by the related antioxidant properties detected by the ABTS and DPPH assays reported below.

**Figure 4:**
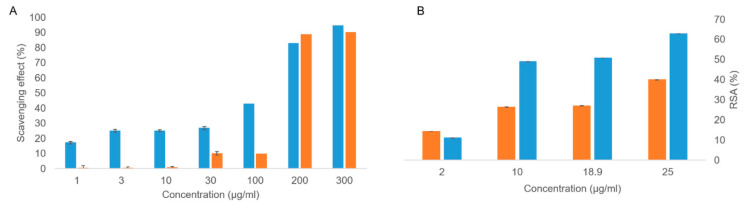
ABTS (**A**) and DPPH (**B**) results of antioxidant activity relevant to OFE (blue bars) and hydroxytyrosol (orange bars).

The ABTS and DPPH antioxidant assays were performed on both the OFE and hydroxytyrosol. As far as the ABTS radical scavenging test is concerned (Figure 4A), the olive extract provided excellent antioxidant activity even at low concentrations (1 μg/mL), with the highest activity at 300 μg/mL (scavenging effect: 94.5%). A high scavenging effect for hydroxytyrosol at 300 µg/mL was also detected (89.9%), although this molecule did not show a gradual improvement in antioxidant activity as the concentration increased but rather an on–off behavior. In the DPPH test (Figure 4B), the olive extract displayed good antioxidant activity, which is proportional to the concentration, in agreement with what was observed by the ABTS test. On the other hand, hydroxytyrosol showed concentration-dependent antioxidant activity, unlike the ABTS test results. Both tests revealed that the OFE antioxidant performances were higher than those observed for hydroxytyrosol, suggesting the presence of different active species in the extract.

### 2.3. Cytotoxicity of OFE Extract

**Figure 5:**
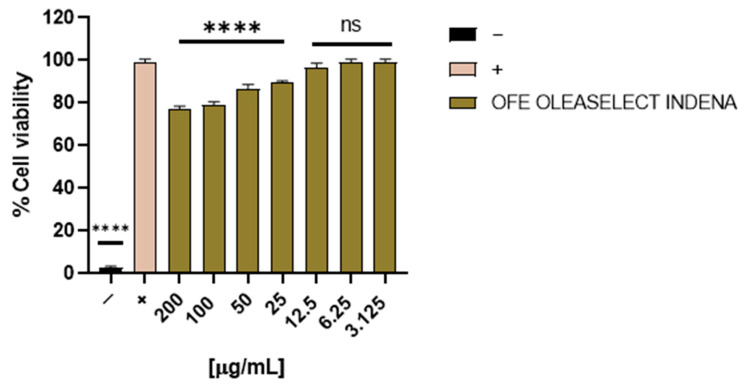
Cytotoxic activity of OFE. Cell viability was evaluated by MTT assay after Vero cell treatment with OFE for 24 h at different concentrations (from 200 to 3.125 μg/mL). Non-treated cells were used as the positive control (+), whereas DMSO was used as the negative control (−). Statistical analyses were determined by one-way ANOVA with Dunnett’s test for multiple comparisons. Significances are referred to as the untreated cells (+). **** *p* < 0.0001; ns: non-significant.

OFE cytotoxicity was evaluated through an MTT assay. In particular, Vero cells were treated with OFE at concentrations ranging from 200 to 3.125 μg/mL for 24 h. After that, cell viability was revealed spectrophotometrically indicating that the extract did not exhibit marked toxicity at the tested concentrations. In addition, slight toxicity was observed starting from 25 μg/mL to 200 μg/mL. However, altogether these data suggested that the OFE could be assessed for its antiviral potential in a broad range of concentrations (Figure 5).

### 2.4. Antiviral Activity of OFE Extract

**Figure 6:**
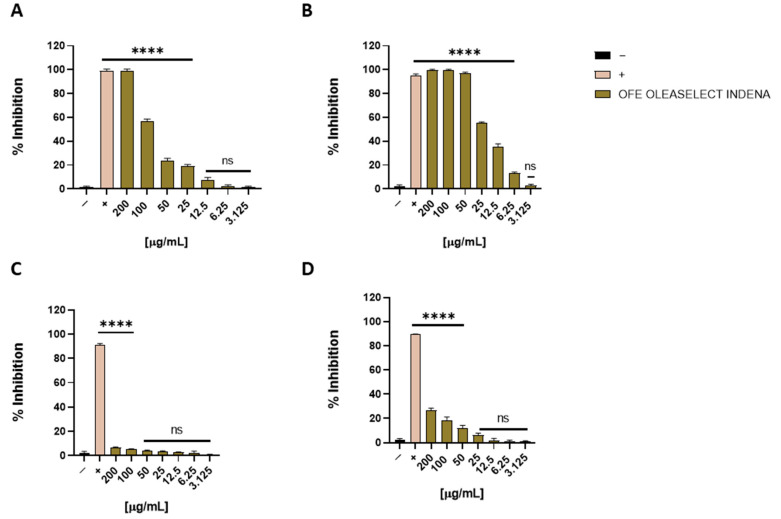
Antiviral activity of OFE against HSV-1. Different assays were performed in order to evaluate anti-HSV-1 activity. (**A**) Co-treatment: simultaneous addition of OFE and virus to the cells; (**B**) Virus pre-treatment: virus incubated with OFE and titrated on the cells; (**C**) Cell pre-treatment: OFE incubated with the cells before the viral infection; (**D**) Post-treatment: OFE added to the infected cells. OFE interfered with the early stages of infection acting in co-treatment (**A**) and virus pre-treatment (**B**) assays, although it was not able to interact with the cellular surface (**C**) or block the viral replication (**D**). Different compounds were used as the positive control (+) for each treatment: Greco grape cane extract [7] (**A**, **B**, 10 μg/mL for both the assays), dextran sulfate (**C**, 1 μM), and acyclovir (**D**, 5 μM), whereas infected cells were used as the negative control (−). Statistical analyses were determined by ANOVA with Dunnett’s test for multiple comparisons. Significances are referred to as the negative control (−). **** *p* < 0.0001; ns: non-significant.

The antiviral activity of the OFE was tested against HSV-1 via a plaque assay. We previously demonstrated that a concentration of 200 μg/mL of OFE completely blocked HSV-1 infection in a co-treatment assay (see Section 2.1). Briefly, Vero cells were treated with the extract and infected at the same time with the virus. To determine the concentration at which the extract could block HSV-1 replication by 50% (IC50), we extended the range of concentrations from 200 to 3.125 μg/mL and repeated the co-treatment test. Figure 6A reported that the IC50 was 80 μg/mL. To better investigate which phase of the HSV-1 lifecycle the extract was able to interfere with, another three plaque assays were performed. They differed by the time at which the OFE was added to the cells and are (i) virus pre-treatment: the virus and extract were first combined and then diluted on the cells; (ii) cell pre-treatment: the cells were pre-treated with the extract and then infected with the virus; and (iii) post-treatment: the cells were first infected with the virus and then treated with the extract. These different assays provided different information about the OFE mechanism of action. In addition, the first two indicate if the extract could intervene in the extracellular phase of infection directly with the virus or the cell membrane, respectively. The last assay can suggest if the extract is able to enter the host cell preventing the intracellular stage of infection. When the virus was pre-incubated with the OFE and then the mixture was inoculated on a Vero monolayer (Figure 6B, virus pre-treatment), a strong improvement in the antiviral activity was observed. Figure 6B shows that the IC50 was 25 μg/mL. On the other hand, the OFE was not active in the cell pre-treatment assay indicating that it did not interact with the cell receptor (Figure 6C). However, a slight inhibition of HSV-1 infection was detected in the post-treatment assay, in particular at the higher concentrations (Figure 6D). Altogether, these data are in agreement with those reported in previous studies on olive leaf extracts [10,12,20] but this is the first study investigating OFE antiviral activity.

### 2.5. Antioxidant Activity of OFE-Loaded Formulations

In this work, three topical formulations were proposed: a hydrogel, an oleogel, and a cream. The antioxidant activity of the three OFE-loaded formulations was evaluated using the same assays carried out for the pure extract. Considering that the amount of the formulation used for both tests was 100 µg/mL, a radical scavenging effect for the hydrogel equal to 64.9 ± 0.3 % and 54.70 ± 0.01 % was found in the ABTS and DPPH assays, respectively. These values evidenced the efficient antioxidant activity of the extract also within the hydrogel. Unfortunately, due to the opacity of the cream- and oleogel-based formulations, it was not possible to quantify the antioxidant activity spectrophotometrically, although a colorimetric variation was visually detected.

### 2.6. Total Polyphenols and Hydroxytyrosol Permeation Evaluation Using a Franz Cell

**Figure 7:**
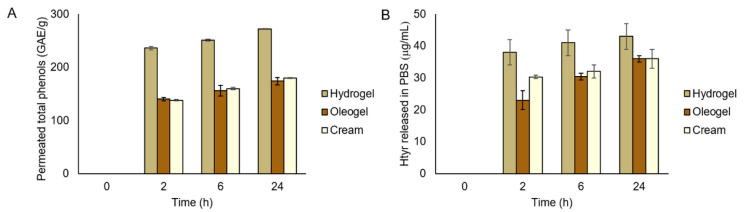
(**A**) Total phenols permeated from OFE-loaded hydrogel, oleogel, and cream across the membrane in Franz cell after 2, 6, and 24h. The evaluation was performed by Folin–Ciocâlteu assay. (**B**) Hydroxytyrosol released directly into the PBS from OFE-loaded hydrogel, oleogel, and cream after 2, 6, and 24h, evaluated by HPLC analysis. All the measurements were performed in triplicate.

The quantification of the total polyphenol and hydroxytyrosol permeation across a skin-model membrane was evaluated using a Franz cell. Usually, the antiviral action of a plant extract is due to the synergistic effect of several components, regardless of the quantity of a single active molecule. To verify the possible transdermal crossing of the organic components present in the three formulations, quantification of both total polyphenols and hydroxytyrosol was carried out following release at 2, 6, and 24 h using the Folin–Ciocâlteu assay and HPLC analysis, respectively. Data are reported in Figure 7. In Figure 7A, it is possible to observe that, in the case of the hydrogel formulation, the number of polyphenols released after 24 h was significantly higher than those observed for the oleogel and cream. This could be related to the high affinity of the hydrogel within the aqueous phase, facilitating the crossing of the membrane and the release of the active substances. Moreover, the number of polyphenols adsorbed on the membrane was also assessed, evidencing amounts of 442 ± 4, 135.0 ± 0.3, and 240 ± 30 GAE/g for the hydrogel, oleogel, and cream, respectively.

Moreover, considering the Franz cell experiments, the amount of hydroxytyrosol in the hydrogel-based OFE formulation was determined by HPLC. HPLC analysis performed after 24 h on the fraction permeated through the Start-M^®^ membrane, revealed that no peak related to hydroxytyrosol was observed. On the other hand, HPLC analysis performed on the Start-M^®^ membranes used for each experiment extracted in the mobile phase showed that the amount of hydroxytyrosol entrapped or adsorbed in the membrane was equal to 2.5 ± 0.1 µg/mL, 0.46 ± 0.02 µg/mL, and 0.45 ± 0.02 µg/mL for the hydrogel, cream, and oleogel, respectively. From these results, it was concluded that hydroxytyrosol transdermal release did not occur efficiently. Therefore, a quantification of the hydroxytyrosol released directly in PBS solution was carried out, as shown in Figure 7B. The hydroxytyrosol release was more efficient from the hydrogel than from the other formulations. All these results were in good agreement with the antiviral performances exerted by the three formulations.

### 2.7. Cytotoxicity of Olive-Based Formulations

**Figure 8:**
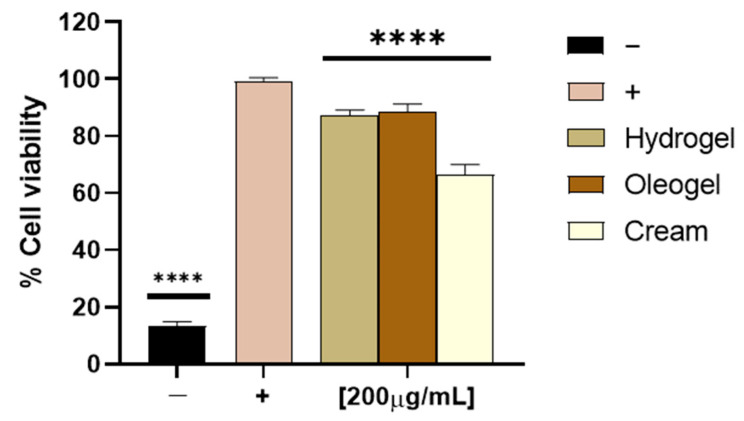
Cytotoxic activity of formulations. Cell viability was evaluated by MTT assay after Vero cell treatment with each olive-derived formulation for 24 h at 200 μg/mL OFE. Non-treated cells were used as the positive control (+), whereas DMSO was used as the negative control (−). Statistical analyses were determined by one-way ANOVA with Dunnett’s test for multiple comparisons. Significances are referred to as the untreated cells (+). **** *p* < 0.0001.

To analyze the cytotoxicity of the hydrogel, oleogel, and cream, we inoculated aliquots of each formulation corresponding to a 200 μg/mL OFE concentration on a Vero cell monolayer. After 24 h, cytotoxicity was revealed spectrophotometrically. The formulations did not exhibit any cell toxicity as shown in Figure 8, except for the cream formulation, which was able to partially affect cell viability.

### 2.8. Antiviral Activity of the Three Formulations against HSV-1

**Figure 9:**
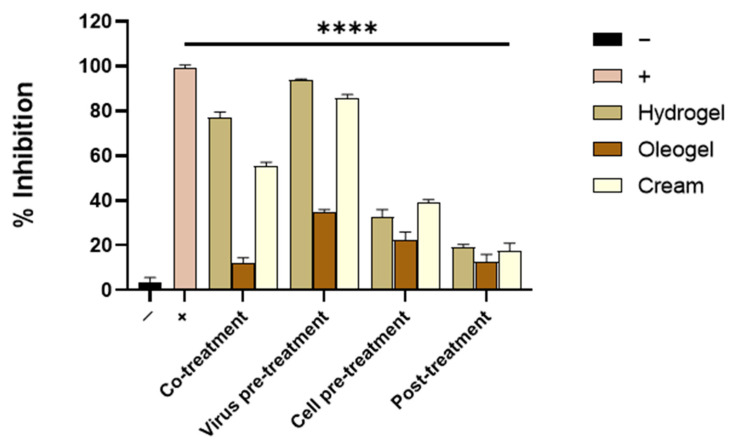
Antiviral activity of OFE formulations against HSV-1. Different assays were performed in order to evaluate anti-HSV-1 activity. Hydrogel and cream formulations interfered with the early stages of infection, acting in co-treatment and virus pre-treatment assays, although they did not exhibit any relevant antiviral activity either in cell pre-treatment or post-treatment tests. Statistical analyses were determined by ANOVA with Dunnett’s test for multiple comparisons. Significances are referred to as the negative control (−). **** *p* < 0.0001.

The three OFE-loaded formulations were evaluated for their antiherpetic potential in vitro. First, Vero cells were incubated together with adequate aliquots of each formulation, containing 200 μg/mL of OFE and HSV-1 in a co-treatment assay (Figure 9). The preliminary results suggested that the oleogel was not active in interfering with HSV-1 infection; meanwhile, the hydrogel and cream formulations exhibited strong antiviral activity. Overall, the hydrogel showed the greatest activity by reducing the herpes replication by 77%, whereas the cream formulation had a minor effect by halving HSV-1 infectivity (55% inhibition). As for the OFE, the antiviral potential of the three formulations has been also investigated in other treatment schemes. To investigate if some of the topical formulations could interfere directly with the viral membrane, a viral pre-treatment test was performed. The results indicated an improved antiviral activity for all the formulations even if oleogel showed a very slight antiherpetic effect. On the other hand, the hydrogel and cream formulations were able to consistently block HSV-1 replication, with 93% and 85% inhibition, respectively. An OFE-free hydrogel, oleogel, and cream were also tested, showing no antiviral activity. This finding suggests that the observed strong antiherpetic activity is specifically due to the olive extract contained in the vehicles. For the last two treatments, i.e., the cell pre-treatment and post-treatment assays, no relevant antiviral effect was observed (Figure 9), demonstrating that none of the three formulations could interact with the infected cell or inside it and that their main action is expressed in the very early phase of infection directly on the viral particles. In addition, these data are very promising suggesting that the hydrogel and cream formulations could be used as topical treatments for both preventing and healing herpes lesions.

### 2.9. Antiviral Activity of the Three Formulations against Acyclovir-Resistant HSV-1

**Figure 10:**
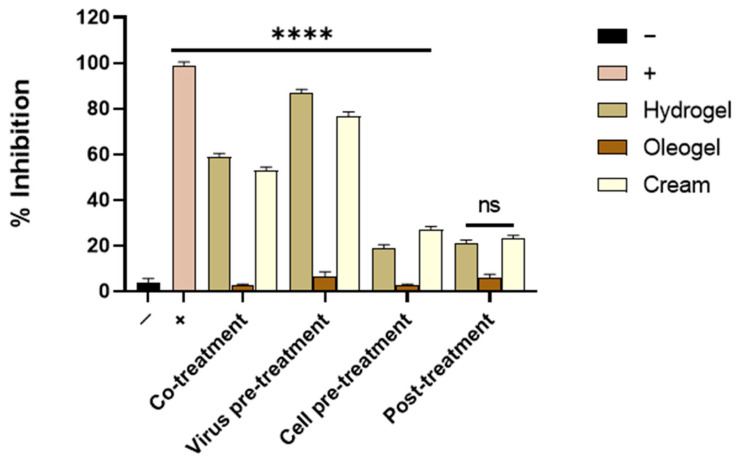
Antiviral activity of OFE formulations against acyclovir-resistant HSV-1. Hydrogel and cream formulations were able to reduce the viral infection by acting in co-treatment and virus pre-treatment assays. Statistical analyses were determined by ANOVA with Dunnett’s test for multiple comparisons. Significances are referred to as the negative control (−). **** *p* < 0.0001; ns: non-significant.

To date, acyclovir is the drug most often used to treat HSV-1 infections [21,22,23]. It can be administered through several routes: (i) topical, by the application of the cream for infections of the skin and mucous membranes; (ii) oral, usually for long-term treatment of mucocutaneous herpes and prophylaxis in immunocompromised patients; (iii) ophthalmic, for herpetic keratitis affecting the eye; and (iv) intravenous, for serious infections in normal and immunocompromised patients. However, the rise and rapid spread of acyclovir-resistant strains have posed a great urgency to search for alternative and valid options and targets for fighting drug-resistant HSV-1. We selected an acyclovir-resistant HSV-1 strain in Vero cells receiving suboptimal acyclovir treatment for a long period. Then, the same set of experiments was performed and the results were successful. Figure 10 indicates that hydrogel and cream formulations were also able to reduce the acyclovir-resistant HSV-1 infection in a very similar way to the parenteral strain.

Kurokawa et al. demonstrated the ability of traditional herbal medicines in combination with acyclovir in inhibiting HSV-1 replication [23]. In addition, when mice were orally co-treated with acyclovir and a herbal extract, there was a progressive regression of skin lesions and the survival times were prolonged. Moreover, a huge number of botanical extracts were studied singularly, as recently reviewed by Garber and coworkers [24]. For instance, *Houttuynia cordata* (*H. cordata*) extract exhibited similar antiviral potential against HSV-1 and the acyclovir-resistant strain (IC50 at 1.11 mg/mL), mainly at the beginning of infection [25]. This mechanism of action is comparable to that we hypothesized for the olive-derived formulations. Furthermore, another important consideration is that the IC50 of our analyzed products was very similar for the parental and mutated strains; therefore, they were able to elicit antiviral effects through a different mechanism than acyclovir. Very recently, other natural bioproducts, i.e., *Rosa* essential oils, have shown a very strong effect in interfering with the viral reproduction of both susceptible and resistant HSV-1 [26]. Here, the proposed mechanism is quite different from that of our formulations since the rose oils, added after the virus entered the cell together with acyclovir at a concentration four times lower than the IC50, considerably reduced the viral yield. Even if many extracts shown in Garber’s review have been shown to be effective against the acyclovir-resistant strain, no olive fruit or leaf extracts have been investigated [24]. Therefore, to our knowledge, the present study highlights for the first time the remarkable antiherpetic potential of both the investigated OFE and the proposed natural formulations. In addition, the hydrogel showed the greatest antiviral effect by acting both in the co-treatment and, particularly, in the virus pre-treatment assay. The fact that the best activity has been observed in the second assay, therefore directly in the viral particle, shows that the topical formulation can act as a therapeutic agent in the very early stages of infection of both wild-type and acyclovir-resistant HSV-1. Its mechanism of action is totally different with respect to the acyclovir and a combinatorial treatment of acyclovir and the olive-derived formulation could be investigated in the future to find a possible synergistic action.

## 3. Materials and Methods

### 3.1. Materials

OFE, botanical origin *Olea europaea* L. (trademark name: Oleaselect^®^), was kindly provided by Indena s.a.s. (France). Oleaselect^®^ is a standardized extract obtained from the fresh pulp of a variety of selected Italian *Olea europaea* that are particularly rich in polyphenols.

For initial screening, five other olive extracts were examined. In particular, another olive fruit extract (Olive powder extract Opextan^®^, Indena) and four OLE (i.e., *Olea europaea* leaves powder extract >6, >12, >20% oleuropein, purchased from Farmalabor, Italy, and >80% oleuropein, Sabinsa Europe GmbH, Langen, Germany) have been taken into account.

Standard hydroxytyrosol for HPLC analysis, gallic acid (GA), 1,1-diphenyl-2-picrylhydrazyl (DPPH), 2,2-azino-bis-(3-ethylbenzthiazoline-6-sulfonic acid) (ABTS), potassium persulfate, sodium carbonate, and Folin–Ciocâlteu’s phenol reagent were supplied by Sigma Aldrich (Milan, Italy). For the hydrogel and cream formulations, pharmaceutical-grade carboxymethyl cellulose sodium salt was supplied by Eigenmann e Vernally S.p.A. (Milan, Italy), whereas the other cosmetic ingredients reported in Section 3.3 were purchased from ZenStore (Salerno, Italy).

### 3.2. Characterization of the OFE

#### 3.2.1. Bulk and Surface Chemical Characterization

FT-IR/ATR analyses using a Spectrum Two-PE instrument endowed with a universal ATR accessory (UATR, Single Reflection Diamond/ZnSe) supplied by PerkinElmer. For each of the relevant samples, FT-IR/ATR spectra were recorded from 400 to 4000 cm^−1^ with a 4 cm^−1^ resolution.

XPS analyses were performed using a scanning microprobe PHI 5000 VersaProbe II purchased from Physical Electronics (Chanhassen, MN). The instrument is equipped with a micro-focused monochromatized AlKα X-ray radiation source. The samples were examined in HP mode with an X-ray take-off angle of 45° (instrument base pressure ~10^−9^ mbar). The size of the scanned area was about 1400 × 200 μm. Wide scans and high-resolution spectra were recorded in FAT mode for each sample, setting pass-energy values equal to 117.4 eV and 29.35 eV, respectively. In order to fit the high-resolution spectra, the commercial MultiPak software version 9.9.0.8 was used. Adventitious carbon C1s were set as the reference charge (284.8 eV).

#### 3.2.2. Thermal Analysis

Thermogravimetric analysis (TGA) is a widely used technique to evaluate the thermal stability of compounds in which the loss of mass as a function of temperature is measured. OFE extracts were examined through a PerkinElmer TGA-400 instrument (PerkinElmer Inc. Waltham, USA). Briefly, 5–10 mg samples were heated in a nitrogen atmosphere in the range of 30–800 °C with a constant flow rate (20 °C/min) and a gas flow set at 20 mL/min. The TGA Pyris series software was exploited to record thermograms (TG) and calculate their respective derivative curves (DTG) for further data mining.

#### 3.2.3. Total Polyphenol Content (TPC) Determination

The TPC in OFE was determined by the Folin–Ciocâlteu colorimetric method as described by Fabiano and coworkers with some modifications [17]. Briefly, for the preparation of gallic acid (GA) stock solution for the calibration curve, 0.5 g of GA were dissolved in 10 mL ethanol and subsequently brought up to 100 mL volume with distilled water, obtaining a concentration of 5 g/L. Suitable dilutions of the GA stock solution were carried out obtaining 100, 150, 250, and 500 mg/L. An amount of 100 μL of each GA solution was added to 7.9 mL of distilled water and 500 µL of Folin–Ciocâlteu reagent. After 8 min, 1.5 mL of sodium carbonate solution (1.9 M) was added and the absorbance was measured spectrophotometrically (UV-visible Spectrophotometer UV-1900i, Shimadzu, Milan, Italy) using a wavelength range from 690 to 810 nm and measuring the absorbance value against the blank at a wavelength of 765 nm. A triplicate of each sample was performed. The TPC in the OFE-based samples was therefore determined by referring to the calibration curve and the results were expressed as mg of gallic acid equivalent to per gram of dry material, i.e., GAE/g. The calibration equation for GA was y = 0.0005x − 0.0325 (R^2^ = 0.9961).

#### 3.2.4. Radical Scavenging Activity by ABTS and DPPH Assays

OFE in vitro antioxidant activity was tested by ABTS and DPPH assays, according to the protocols described by Luo et al. [27].

Briefly, ABTS was dissolved in PBS (0.01 M, pH 7.4) at a concentration of 7 mM and the radical cation was obtained after 16 h of reaction in the dark with 2.45 mM ammonium persulfate (APS) and then was diluted to absorbance 0.70 ± 0.02 at 734 nm before use. An amount of 0.2 mL of the sample (in the range 1–1000 μg/mL) was mixed with 2.0 mL of ABTS^+^ and the absorbance was measured at 734 nm after 6 min. The antioxidant activity was calculated using the following equation:

ABTS scavenging effect (%) = [A_0_ − (A_s_ − A_b_)]/A_0_ × 100

where A_0_ is the ABTS^+^ absorbance, A_s_ is the absorbance of ABTS^+^ with the sample, and A_b_ is the absorbance of the sample without the radical cation.

As far as the DPPH test is concerned, DPPH solution 100 µM was prepared in methanol and its absorbance was measured at 517 nm. Calibration curves (r2 = 0.999) were obtained with OFE standard solutions (2 to 25 ppm). An amount of 3 mL of standard or sample solution was mixed with 1 mL of DPPH solution and their absorbance was measured at 517 nm. The radical scavenging activity percentages (% RSA) were calculated with the following equation:

%RSA = (A_rad_ − A_S_)/A_rad_ · 100

in which A_S_ represents the sample’s absorbance, whereas A_rad_ is the absorbance of the bare DPPH or ABTS. Each measurement was performed in triplicate and expressed as mean ± standard deviation. All the assays were performed using a UV-visible Spectrophotometer UV-1900i, (Shimadzu, Milan, Italy). Ascorbic acid (50 µM) scavenging activity on DPPH and ABTS radicals was also assessed and compared to the extract’s activity.

#### 3.2.5. HPLC Analysis

OFE was analyzed by HPLC (Prominence Series 20 with SPD-M20A PDA detector, Shimadzu, Milan, Italy) for hydroxytyrosol content, adapting the method previously described by Tamasi et al. [13], after a preliminary extraction in ethyl acetate and filtration with PTFE filters with a diameter of 0.45 µm. Each sample was tested in triplicate and data were reported as mean ± standard deviation. A Shim-Pack GIST C18-AQ column (150 mm × 4.6 mm, 5 µm Shimadzu) was eluted in isocratic mode at 30 °C, 30% acetonitrile, and 70% water. The effluent was monitored at 230 nm. The mobile phase flow rate was kept at 1 mL/min and samples were injected through a 20 µL injection loop. LabSolutions software was exploited to build a calibration curve (r2 0.999) with the standard compound dissolved in the mobile phase at four concentrations (1, 5, 25, 100 µg/mL).

### 3.3. Preparation of the OFE Formulations

To convey the plant active ingredients on the skin, three types of formulations were developed: a water-based gel formulation (called hydrogel), an oil-based gel formulation (called oleogel), and a cream. As for the gel-based formulations, they could be categorized as either hydrogels or oleogels. A hydrogel contains water as a liquid component and a hydrogelator, such as a polyacrylate (in traditional formulations) or a natural gum (in eco-biological formulations), whereas an oleogel is composed of an emollient oil, which can be mineral oil or a synthetic ester (in traditional formulations) or a vegetal triglyceride (in eco-biological cosmetics) and an oleogelator (usually micronized silica), which entraps the oil to form a complex semisolid structure [28]. Finally, a cream is a formulation characterized by the emulsion of an aqueous phase and an oily phase. The compositions of the formulations are reported in Table 1. The preparation procedure for the gels consisted of one-step mixing at room temperature; as far as the cream preparation is concerned, two phases (i.e., aqueous and lipophilic) have been prepared separately and heated at 65–70 °C. Then, a water-in-oil emulsion was performed, and the active principle and the preservative were added at the end of the emulsion at 45 °C. The pH of the final formulations was 5.50 ± 0.02.

### 3.4. Cell Lines and Virus

Vero cells (ATCC CCL-81, Manassas, Virginia, United States) were cultivated in Dulbecco’s Modified Eagle Medium (DMEM) with 4.5 g/L glucose (Microtech, Naples, Italy) supplemented with a penicillin/streptomycin solution (Himedia, Naples, Italy) and 10% Fetal Bovine Serum (FBS, Microtech). HSV-1 (strain SC16), containing a lacZ gene driven by the cytomegalovirus IE-1 promoter to express beta-galactosidase, was grown on a Vero cell monolayer, as previously reported [29]. An acyclovir-resistant HSV-1 strain was selected in Vero cells receiving suboptimal acyclovir (ACV, Sigma-Aldrich) treatment.

### 3.5. Cytotoxicity Tests

Vero cells were seeded at 5 × 10^4^ cells/mL in a 96-well plate and incubated with several concentrations of each extract ranging from 3.125 to 200 μg/mL or with each olive-derived formulation. After 24 h, all wells were treated with 100 μL 3-(4,5-Dimethylthiazol-2-yl)-2,5-Diphenyltetrazolium Bromide (MTT, Sigma-Aldrich, St. Louis, MO, USA) solution (5 mg/mL) and incubated at 37 °C/5% CO_2_ for 3 h. Then, 100 μL DMSO (Sigma-Aldrich) was added to each well to dissolve the purple formazan, and absorbance was measured at 570 nm. An amount of 100 μL of DMSO was used for the negative control (CTRL −), and 100 μL of culture medium represented the positive control (CTRL +).

### 3.6. Antiviral Activity

Vero cells were plated (2 × 10^5^ cells/well) in 24-well plates the day before the experiment. Then, a set of experiments were performed [6]: (i) a co-treatment assay: Vero cells were treated at the same time with non-cytotoxic concentrations of OFE extract and the virus at 0.01 multiplicity of infection (MOI) for 1 h at 37 °C; (ii) virus pre-treatment: virus (MOI 0.1) was treated with extract for 1 h at 37 °C and then the mixture was diluted on a cell monolayer for a further hour at 37 °C; (iii) cell pre-treatment: cells were pre-treated with the extract for 1 h at 4 °C and then they were infected with the virus (MOI 0.01) and shifted at 37 °C; and (iv) post-treatment: cells were first infected with the virus (MOI 0.01) for 1 h and then treated with extract for another hour at 37 °C. For the olive formulations (hydrogel, oleogel, and cream), the same experimental procedure was carried out using aliquots corresponding to a 200 μg/mL OFE concentration. Moreover, OFE-free hydrogel, oleogel, and cream were also tested. At the end of each treatment, Vero cells were washed with Phosphate Buffered Saline (PBS, Microtech) and overlaid with DMEM supplemented with 5% carboxymethyl cellulose (CMC, Sigma-Aldrich, St. Louis, MO, United States) for 48 h. Finally, cells were fixed and stained with 4% formaldehyde (Sigma-Aldrich) and 0.5% crystal violet (Sigma-Aldrich), respectively, and the viral plaques were counted. The percentage of viral inhibition was calculated in relation to non-treated cells (CTRL −), as follows:

% viral inhibition = (100 − (plaques counted in the test sample)/(plaques counted in the negative control)) × 100(1)

### 3.7. In Vitro Skin Permeation Studies

A jacketed Franz diffusion cell (PermeGear Inc., SES GmbH, Bechenheim, Germany) was exploited to assess in vitro skin permeation of OFE eluting from each formulation, following the protocol reported in a previous work [30]. Briefly, the formulations were placed in the cell donor compartment. An O-ring joint kept the OFE formulation (1 g) on the synthetic StratM^®^ membrane (Merck KGaA, Darmstadt, Germany), characterized by skin-like porosity, diffusivity, and composition. The whole assembly was fixed with a stainless-steel clamp to maintain the tight connection between the donor and receptor compartments. The Franz cell receptor chamber was filled with 5 mL of PBS (pH 7.4) and continuously stirred on an ATE magnetic stirrer (VELP Scientifica Srl, Usmate, Italy). The temperature was kept constant at 32.00 ± 0.03 °C with a CD-B5 heating circulator bath (Julabo GmbH, Seelbach, Germany). At predetermined time points (2, 6, and 24 h), PBS aliquots of 500 µL were withdrawn, replaced with fresh PBS, and analyzed by HPLC (Prominence Series 20 with SPD-M20A PDA detector, Shimadzu, Milan, Italy) for hydroxytyrosol content, as described in the HPLC Analysis Section. Additionally, at the end of the experiments, the Strat-M^®^ membrane was placed in the HPLC mobile phase overnight at 25 °C to extract and quantify the hydroxytyrosol retained by the membrane. TPC retained by the membrane was also assayed by the Folin–Ciocâlteu colorimetric method.

### 3.8. Statistical Analysis

Each antiviral test was performed in triplicate and expressed as mean ± Standard Deviation (SD). One-way ANOVA was followed by the Dunnett’s multiple comparisons test and all the graphs were generated by GraphPad Prism (version 8.0.1; Software for 2D graphing and statistics; GraphPad Software Inc.: San Diego, CA, USA, 2018).

## 4. Conclusions

In this work, a detailed study of the physical–chemical and biological properties of an OFE, particularly rich in polyphenols, was carried out, evidencing its suitability to be employed as a natural antiviral agent against HSV-1. Moreover, a hydrophilic (a hydrogel), a lipophilic (an oleogel), and a system containing both hydro- and lipo-phases opportunely emulsified (a cream) were developed to convey this natural extract, with a particular focus on the realization of completely ecological formulations. It was ascertained that all three formulations exerted their main activity in the early phases of herpetic infection by covering the viral particles and hampering subsequent entry into the host cells. Finally, the permeation tests of the active ingredient simulated by the Franz cell and the antiviral activity results converged towards the selection of the OFE-loaded hydrogel formulation as the best choice to fight against HSV-1 infection, including the acyclovir-resistant strain.

## Figures and Tables

**Table 1:** Composition of the three formulations.

*Ingredients*	*Percentage (%)*		
	*Hydrogel*	*Oleogel*	*Cream*
Water	92.4%	--	70%
Glycerol ^1^	5%	--	3%
Carboxymethyl cellulose ^2^	1%		0.4%
Avocado oil ^3^	--	90.5%	6%
Micronized silica	--	8%	--
α-Tocopherol	--	0.5%	--
Coconut oil ^3^	--	--	6%
Cocoa butter	--	--	6%
Glyceryl monostearate SE	--	--	5%
OFE	1%	1%	1%
Preservative (ecocert ^4^)	0.6%	--	0.6%

^1^. Vegetal origin. ^2^ Pharmaceutical-grade. ^3^ Biological origin. ^4^ Benzyl alcohol and dehydroacetic acid.

## Data Availability

The data presented in this study are available herein.

## Sample Availability

Samples of the natural extracts, standard compounds, and topical formulations are available from the authors.
